# Moyamoya Disease From an Otolaryngologist’s Perspective: A Rare Case

**DOI:** 10.7759/cureus.28108

**Published:** 2022-08-17

**Authors:** Gajanan Chavan, Aparna Chavan, Govind Nagdev, Gaurang M Aurangabadkar

**Affiliations:** 1 Emergency Medicine, Datta Meghe Institute of Medical Science, Wardha, IND; 2 ENT, RKDF Medical College, Bhopal, IND; 3 Emergency Medicine, Datta Meghe Institute of Medical Science Wardha, Wardha, IND; 4 Respiratory Medicine, Datta Meghe Institute of Medical Science, Nagpur, IND

**Keywords:** facial palsy, magnetic resonance angiography, otolaryngologist, down's syndrome, moya moya disease

## Abstract

Moyamoya disease is characterized by narrowing of cerebral blood vessels and is progressive in nature. We present the case of a 21-year-old female patient who was a known case of Down’s syndrome and presented with right-sided facial muscle weakness and was initially suspected of having cholesteatoma, but no evidence for the same was found after extensive radiological investigations. The suspicion of a central nervous system pathology was raised due to the clinical findings of dysphasia and supranuclear facial palsy. Magnetic-resonance angiography (MRA) was suggestive of findings of early Moyamoya disease. After a Neurologist consultation, the patient was started on conservative management with anti-platelet drugs and Statins and had symptomatic improvement. The patient was advised regular follow-up and had no fresh episodes of similar complaints.

## Introduction

Moyamoya disease (MMD) is a rare progressive vascular disorder that is characterized by the narrowing of vessels in the brain. Children and young adults are most commonly affected [1,2]. Moyamoya is the Japanese term used for something hazy, just like a puff of cigarette smoke drifting in the air, and was coined by Suzuki and Takaku in 1969 [2]. This haziness or puff of smoke refers to the radiographic appearance of collaterals developed at the base of the brain in this disease. This disease has several unique features [3]. Although the cases are seen all over the world the highest incidence is seen in East Asia which is 2.3 and 0.94 per 100,000 individuals in South Korea and Japan respectively. Women were seen to be affected more than males [4,5]. Clinically this disease can be represented as ischemic or hemorrhagic stroke. For isolated aphasia or facial nerve palsy, though a rare clinical presentation, otolaryngologists may be the first point of contact. Hence, MMD becomes one of the “must know” facts for otolaryngologists. The bimodal age distribution is one of the characteristic features [4,5]. Age at presentation affects the clinical characteristics; in young people, ischemic symptoms are more prevalent, whereas the risk of hemorrhage rises with advancing years. Clinical signs in children with MMD range from transient ischemic attack (TIA) to lifelong neurological impairments, including headache, seizures, involuntary movements, and sensory and motor deficiencies. High clinical suspicion is necessary for the diagnosis, which can be verified by cerebral angiography and magnetic resonance angiography (MRA). Etiopathogenesis of MMD in Down’s syndrome (DS) is sparse. The most prevalent chromosomal defect (trisomy 21) causing mental impairment in both sexes is DS.

## Case presentation

A 21-year-old female patient with DS presented with the onset of right-sided facial paresis. On examination, right-sided facial paresis was confirmed. On examination, keratin debris was also seen in the right ear raising the suspicion of cholesteatoma. After cleaning the keratin debris, otoscopy revealed attic retraction. Hence, HRCT temporal bone was done suspecting cholesteatoma. It did not reveal any middle ear disease except for the thickening of the tympanic membrane. The patient was sent with antibiotics and steroids and topical ear drops with the provisional diagnosis of Bell’s palsy. Eventually, after two days she developed dysphasia. The patient had proper phonation but lacked intelligent and clear speech.

**Figure 1:**
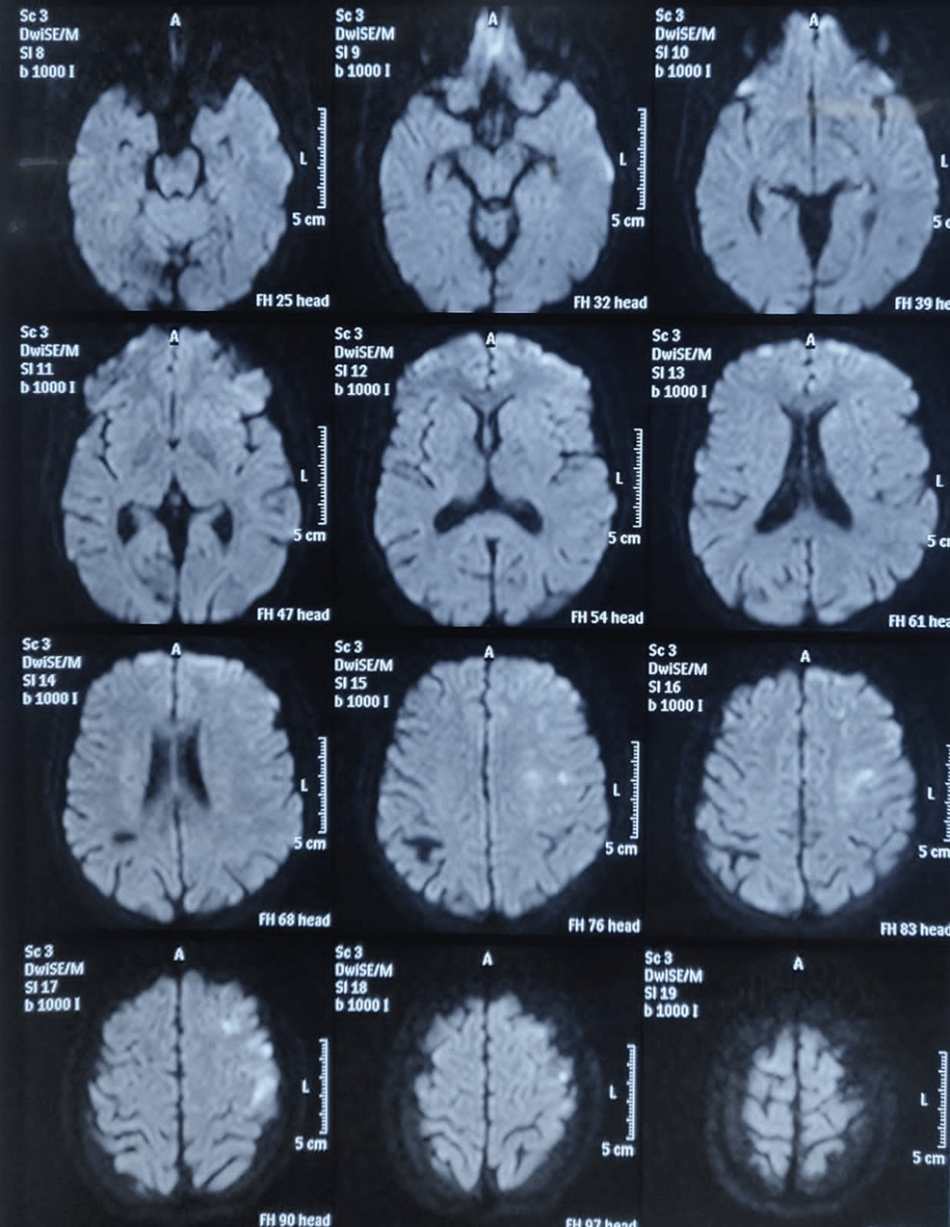
Magnetic resonance imaging (MRI) of the brain showing multiple restrictions in the left frontal as well as high parietal region suggestive of multiple non-hemorrhagic infarcts

The facial nerve was partially recovered now involving only the angle of the mouth and eye closure. The patient was again thoroughly and carefully examined. Supranuclear facial palsy and dysphasia raised the suspicion of central pathology. Probably the pathology of the right ear was a coincidental finding. In ear pathology, facial palsy has to be infra-nuclear and not supranuclear. Secondly, in a right-handed person, cholesteatoma in the left ear and not the right ear will present with dysphasia/aphasia, as Broca’s area is on the dominant left temporal lobe. Hence MRI brain was advised that revealed multiple brain infarcts in the frontal region (Figure 1).

**Figure 2:**
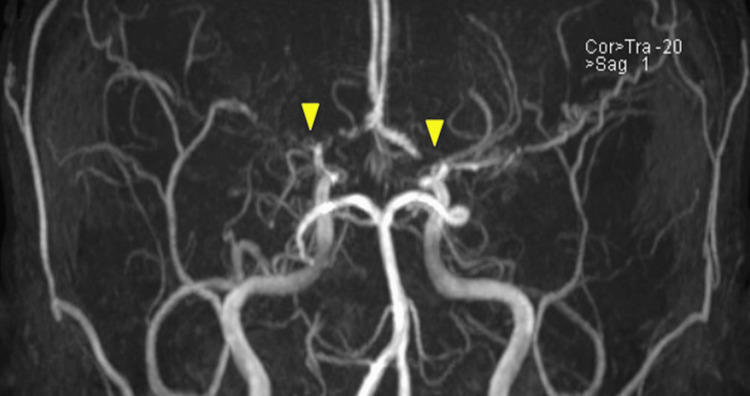
Magnetic resonance angiography (MRA) of the brain showing findings of occlusion in the origin of the left riddle cerebral artery (MCA) and severe stenosis and occlusion of the right MCA

A neurologist was consulted who advised CBC, lipid profile, Hb electrophoresis, and coagulation profile. MR angiography was advised which showed radiological features of MMD (Figure 2).

**Figure 3:**
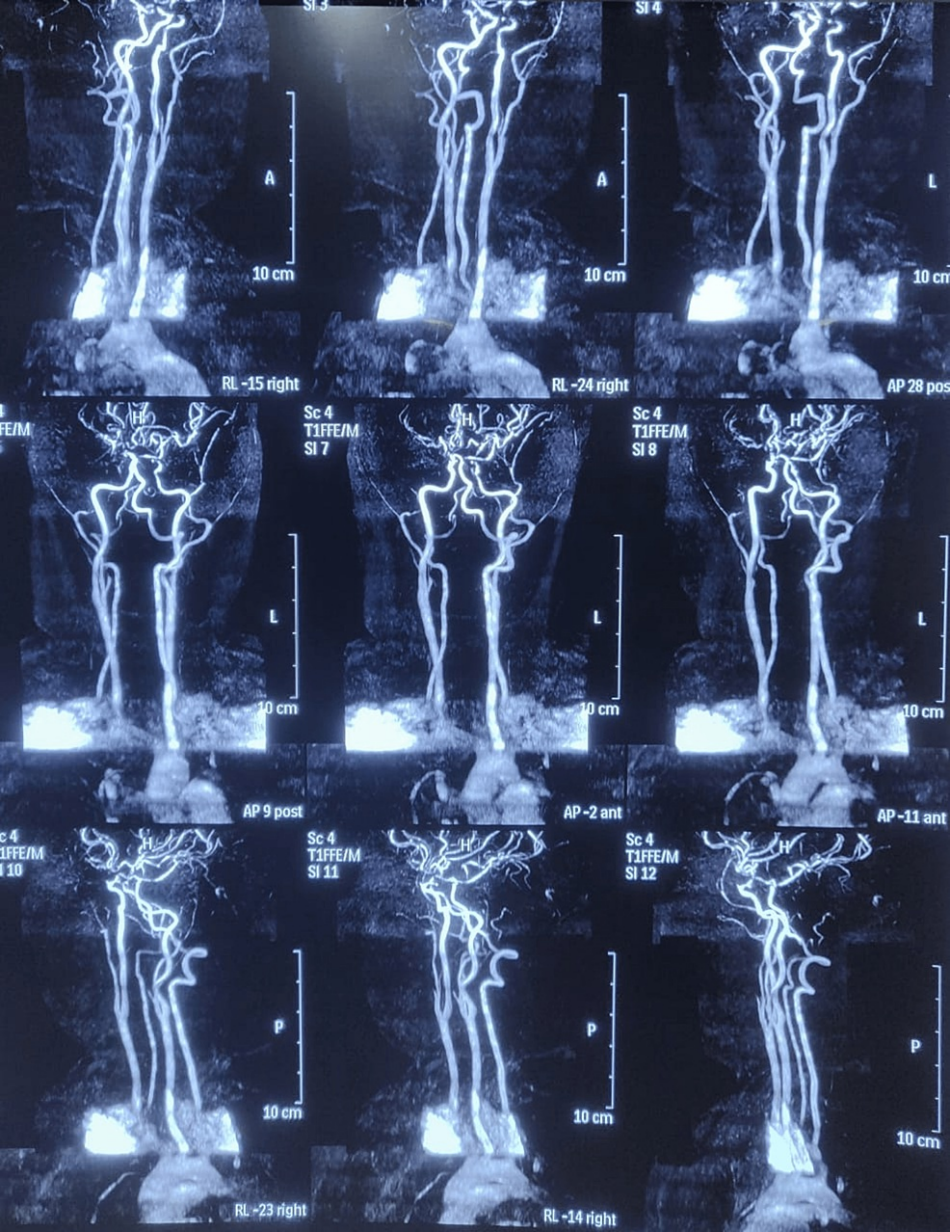
MRI of the neck and cerebral angiography showing severe narrowing of bilateral supra-clinoid internal carotid arteries (ICA), bilateral middle cerebral arteries (MCA) and right posterior cerebral artery (PCA) showing significant luminal narrowing in the proximal segment

MRI neck and cerebral angiography were also done which was suggestive of diffuse narrowing of bilateral infra-clinoid internal carotid arteries (ICA) and severe narrowing of the supra-clinoid ICA and Middle cerebral arteries (MCA) and findings were reported to be suspicious of early MMD (Figure 3).

Since this was the first episode, she was managed conservatively. The patient is still in follow-up for the last eight months with no recurrences. Hence the procedure of pial synangiosis is still under consideration. She is currently on aspirin and atorvastatin treatment daily.

## Discussion

MMD may not be new to neurologists or a neurosurgeon but many Otolaryngologists may be unaware of MMD. In day-to-day clinical practice, Otolaryngologists often come across cases of facial nerve palsy/paresis and aphasia/dysphasia with otogenic in origin. Facial nerve palsy is often infra-nuclear and many times it has been found to be idiopathic. It is extremely rare for a patient with MMD to present to an otolaryngologist. Although diagnosing MMD can be difficult, a typical presentation, a high index of suspicion, and the right imaging study can help us make an accurate and quick diagnosis. MMD is a rare disease found with varying frequency in different geographical regions. MMD is characterized by chronic and progressive stenosis of distal internal carotid arteries and its terminal branches anterior and middle cerebral artery [5]. East Asia has the largest incidence of cases, and women are more commonly affected than men. According to epidemiological research, the prevalence of MMD is 2.3 and 0.94 per 100,000 people, respectively, in South Korea and Japan, respectively [1,5]. In spite of the fact that the majority of cases are sporadic, 10%-15% of patients have a family history of MMD. Although the cause is unknown, investigations suggest that chromosome 17 may be involved [1,6].

MMD can have multiple presentations, partly influenced by age and geographical region [5]. The clinical signs may be categorized mainly into two types: ischemic stroke due to cerebral ischemia and hemorrhagic stroke. Presentations vary between pediatric and adult patients. The prevalence of these two types of symptoms varies across adult and pediatric patients. The majority of pediatric patients have progressive cerebral ischemia, including cerebral infarctions and transient cerebral ischemic episodes. The initial sign-in youngsters may be mental impairment or convulsions. Intracranial bleeding is the initial symptom in half of the adult cases, and ischemic symptoms are the initial sign in the other half [1].

Trisomy 21-related DS is a typical cause of mental disability. In India, 20,000 infants are born each year with DS. Children with DS are more likely to experience cerebral infarction, with the most prevalent reasons being thromboembolism brought on by anomalies in the atrioventricular canal, right-to-left shunting, cardiac valve abnormalities, or myocardial dysmotility. Patients with DS can also develop stroke for various reasons, one of which is MMD [7].

See et al. in their study [8] compared the surgical outcome of MMD in DS with that of the general population. This study stated that the average age of diagnosis was higher with DS suggesting a possible delay in reaching a correct diagnosis of the cause of cerebral ischemia. Pial synangiosis provided long-term protection from stroke in all patients treated.

The gold standard investigation is cerebral angiography [4,5]. Positron emission tomography (PET) and cerebral blood flow: single photon emission computed tomography (CBF-SPECT), both have been utilized successfully in the assessment of cerebral blood flow (CBF), which has significant implications in diagnosing and staging the severity of cerebral ischemia/infarct in MMD (ischemic type) [5,9]. Low CBF is also a sign that revascularization surgery is necessary [9]. When hyperventilation stops, electroencephalography reveals the appearance of Delta waves, which are a characteristic observation in MMD [1].

Currently, there is no proof that pharmacological therapy can stop the progression of MMD or even slow it down. The only clinical symptoms of MMD that are currently being treated with medications, including ischemia and hemorrhage by anti-coagulant or hemostatic actions [4].

Although aspirin is advised for secondary stroke prevention but assessing the patient for surgical revascularization should be a priority [1]. Surgical treatment revolves around direct, indirect, and combined (direct plus indirect) revascularization procedures [1,5] and is recommended for recurrences and low blood flow cases. Various studies show that the incidence of recurrence of intracranial bleed, TIA, mortality, and severe disability was less in the revascularization group as compared to the conservatively treated group.

## Conclusions

MMD is a rare and progressive cerebrovascular disease. In cases of supranuclear facial nerve palsy and Aphasia/dysphasia otolaryngologist should be aware of MMD as one of the differential diagnoses. Thorough clinical examination, clinical correlation, a high index of suspicion, and timely referral can prevent mortality and morbidity in rare disorders. Otolaryngologists rarely see patients with MMD in their routine clinical practice and findings of supranuclear facial palsy along with abnormalities of phonation and speech should raise the possibility of this rare diagnosis.
